# Identification of a Hypoxia-Related Signature for Predicting Prognosis and the Immune Microenvironment in Bladder Cancer

**DOI:** 10.3389/fmolb.2021.613359

**Published:** 2021-05-07

**Authors:** Minxiao Jiang, Liangliang Ren, Yuanlei Chen, Huan Wang, Haiyang Wu, Sheng Cheng, Gonghui Li, Shicheng Yu

**Affiliations:** Department of Urology, Sir Run-Run Shaw Hospital, Zhejiang University School of Medicine, Hangzhou, China

**Keywords:** bladder cancer, hypoxia, signature, disease-specific survival, immune microenvironment

## Abstract

Accumulating evidence indicates that hypoxia is highly associated with bladder cancer genesis, progression, and immune microenvironment. Nevertheless, few studies have identified the role of hypoxia-related genes as a prognostic signature in bladder cancer. This study aimed to establish a hypoxia-related signature with high accuracy for prognosis and immune microenvironment prediction in bladder cancer. We obtained expression profiles and clinical information from Gene Expression Omnibus and The Cancer Genome Atlas. Then the univariate Cox regression, random survival forest algorithm, and multivariate Cox regression analysis were conducted to identify the core genes and four hypoxia-related genes (ANXA2, GALK1, COL5A1, and HS3ST1) were selected to construct the signature. Kaplan-Meier survival analysis demonstrated that patients with a low-risk score had a higher disease-specific survival rate (*p* < 0.0001). The areas under the curve of the signature were 0.829 at 1 year, 0.869 at 3 years, and 0.848 at 5 years, respectively. Additionally, we found this hypoxia-related signature was highly correlated with tumor immune microenvironment and had the potential to predict the efficacy of immunotherapy. In summary, our study developed a hypoxia-related signature, which had high accuracy for prognosis prediction and the potential to guide the immunotherapy for bladder cancer patients.

## Introduction

Bladder cancer is the most frequent cancer in the urinary system, which is estimated to have 81,400 new diagnosed cases and 17,980 deaths in the United States in 2020 (Siegel et al., 2020). There are two subtypes of bladder cancer: approximately 75% of patients are non-muscle-invasive bladder cancer (NMIBC) (Babjuk et al., 2019), and the others are muscle-invasive bladder cancer (MIBC) (Witjes et al., 2020). Unlike many other cancers, the diagnostics, treatments, and five-year survival rates for bladder cancer have been unchanged for 3 decades. Bladder cancer remains a great threat to human health worldwide with a recurrence rate of 60–70% (Berdik, 2017). Therefore, it is valuable to develop a signature that has high accuracy and great efficacy for predicting the prognosis and guiding the treatment for bladder cancer patients.

Tumor microenvironments (TME), such as hypoxia, have attracted great attention in most solid tumors (Brahimi-Horn et al., 2007). Previous studies have found hypoxia microenvironments played an important role in progression, metastasis, and angiogenesis in bladder cancer (Reiher et al., 2001; Luo et al., 2019; Su et al., 2019). Immune feature is another vital feature and immunotherapy has shown great potential in treatment for bladder cancer (Kamat et al., 2017; Koshkin and Grivas, 2018; Marshall and Djamgoz, 2018). Interestingly, there has been increasing evidence indicating an association between the tumor hypoxia feature and tumor immune suppression and immune escape. Hypoxia microenvironment can reduce the natural killing capability in NK cells (Balsamo et al., 2013b). A low oxygen environment may significantly alter cytolytic T lymphocyte development (Caldwell et al., 2001). Moreover, the expression of PD-L1 has a correlation with the expression of HIF-1α (Koh et al., 2019). Hence, the hypoxia feature may be utilized as a biomarker to predict the immune microenvironment (IME) and the efficacy of immunotherapy.

In the present study, we systematically integrated a series of cohorts available provided on the online database and identified four hypoxia-related genes that were highly correlated with the survival status of the bladder cancer patients. Then a novel signature and a nomogram were developed for further survival evaluation. Moreover, we investigated the potential association between the hypoxia-related signature and IME.

## Materials and Methods

### Data Collection and Processing

**FIGURE 1:**
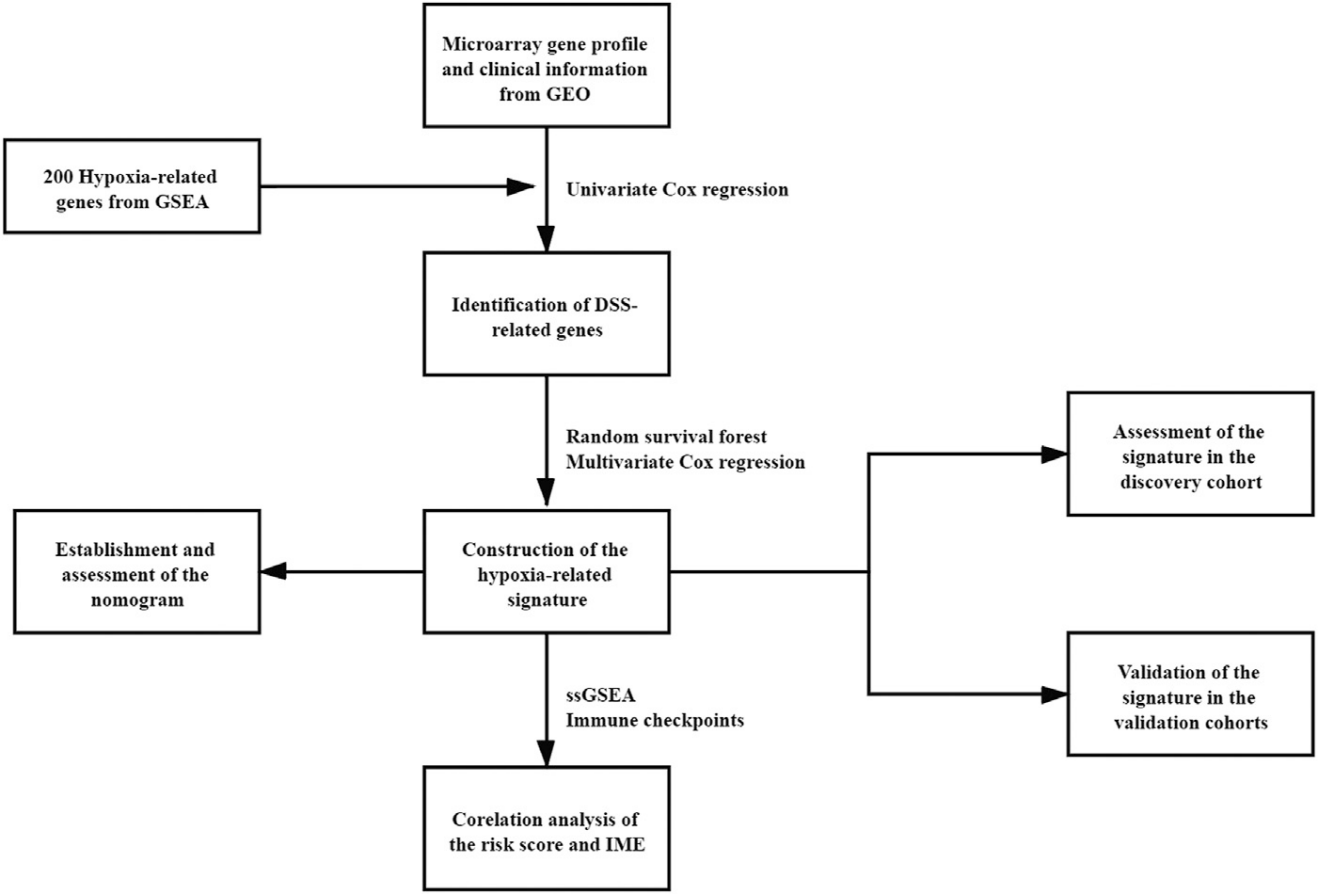
The workflow chart of the study.

Four microarray cohorts based on the Illumina platform, GSE32894, GSE32548, GSE13507, and GSE48075 (Kim et al., 2010; Lee et al., 2010; Lindgren et al., 2012; Sjodahl et al., 2012; Choi et al., 2014; Guo et al., 2020), were obtained from the Gene Expression Omnibus (GEO, https://www.ncbi.nlm.nih.gov/geo/) database. One RNA-sequencing cohort, TCGA-BLCA, was downloaded from The Cancer Genome Atlas (TCGA, https://portal.gdc.cancer.gov/) by the package “TCGAbiolinks”. Then, the microarray raw data were normalized by the package “limma” and probes unexpressed were filter out (Xie et al., 2009; Ritchie et al., 2015). The TCGA-BLCA raw data were normalized by the package “DESeq2” (Love et al., 2014). For all cohorts in this study, only patients with available expression profiles and complete disease-specific survival (DSS) or overall survival (OS) information were included for further analyses. GSE32894 contained 224 bladder cancer patients was chosen as the discovery cohort. Other cohorts were used for external validations. GSE32548 contained 130 primary bladder cancer patients, GSE13507 contained 165 primary bladder cancer patients, GSE48075 contained 73 primary bladder cancer patients and TCGA-BLCA contained 394 bladder cancer patients. Detailed clinical characteristics of each cohort were provided in Table S1-5. The hypoxia-related gene set was obtained from the Gene Set Enrichment Analysis database (GSEA, http://software.broadinstitute.org/gsea/index.jsp). The workflow chart of this study was shown in Figure 1.

### Establishment and Validation of the Hypoxia-Related Signature

Univariate Cox regression analysis was performed to identify the genes associated with DSS and genes with a *p*-value <0.01 were defined as the core genes. Then, the random survival forest algorithm was used to sort the core genes *via* the package “randomForestSRC”. The parameters used in this algorithm were nsplit = 10, nrep = 100, k = 5, nstep = 5 (Gilhodes et al., 2017). Finally, a risk score was calculated for each patient according to the following formula built by multivariate Cox regression:

$$risk\;score=\overset{n}{\underset{i=1}{\sum }}\;\beta_{i}\;*\;Exp_{i}$$

All patients were separated into two different groups according to the median risk score. Time-dependent receiver operating characteristic (ROC) analysis and Kaplan-Meier survival analysis were operated to test the prediction accuracy and ability of this signature.

### Construction and Assessment of the Nomogram

The nomogram was constructed to predict the 3-years and 5-years DSS rate based on the hypoxia-related signature and clinical characteristics *via* the package “rms” (Iasonos et al., 2008). Calibration curves were used to assess the predictive accuracy of the nomogram and the 45° line in the calibration curve represented the actual value.

### Gene Set Enrichment Analysis

The Gene Ontology analysis (GO) (Gene Ontology, 2006) and the Kyoto Encyclopedia of Genes and Genomes (KEGG) (Kanehisa and Goto, 2000) were utilized to identify the main signaling pathways enriched in different risk groups (Subramanian et al., 2005). For each analysis, significant enriched pathways were considered to have a *p*-value < 0.05.

### Evaluation of the Correlation Between the Signature and Immune Microenvironment

We estimated the relative abundance of the immune cells in the GSE3289 cohort *via* the single-sample gene set enrichment analysis (ssGSEA) algorithm. The marker gene set for 28 immune cell types was obtained from previous research (Charoentong et al., 2017). We also applied the ESTIMATE algorithm for the assessment of the abundance of stromal cells and immune cells and calculated the ImmuneScore, StromalScore, and ESTIMATEScore for each patient in the discovery cohort (Yoshihara et al., 2013). To further explore the underlying correlation between the hypoxia-related signature and the efficacy of immunotherapy, we chose some important immune checkpoints to infer the immunotherapeutic response and investigated the expression of these immune checkpoints in different risk groups.

### Cell Culture and Validation of Hub Gene Expression *via* Quantitative Real-Time PCR

Human bladder cancer cells (TCC and J82) were purchased from the American Type Culture Collection (ATCC, Manassas, VA, United States). The TCC and J82 cells were cultured in Minimum Essential Medium (MEM, Corning) with 10% fetal bovine serum (FBS, BI) at 37°C in 5% CO_2_.

To validate the hub gene expression in the hypoxia environment, the TCC and J82 cell lines were cultured at 37°C in 1% O_2_ for 24 h. Then, total RNA was extracted by using TRIzol reagent (Invitrogen, Waltham, MA) and was stored at −80°C. Reverse Transcription System (Promega, Madison, WI) was used for cDNA synthesis according to the protocol provided by manufacturer. The mRNA expression levels of hub genes were measured by qRT-PCR using the ABI PRISM 7500 Sequence Detector System (Applied Biosystems, Waltham, MA), and were normalized to the expression of β-actin RNA. PCR primers used were list in Table S6.

### Immunohistochemistry Experiments

The bladder cancer tissues were fixed in 4% neutral-buffered paraformaldehyde, embedded in paraffin, cut into 5-μm sections and used for immunohistochemistry (IHC) experiments. After Deparaffinizing and rehydration, the sections were treated with 3% H_2_O_2_ for 10 min and citrate buffer at 98°C for 3 min to enhance antigen exposure. Then the sections were incubated overnight with the primary antibodies ERBB3 (HuaBio, China), FGFR3 (HuaBio, China), CTLA4 (Bioss, China), CD274 (PD-L1) (HuaBio, China), PDCD1 (PD-1) (HuaBio, China), and LAG3 (abcam, United Kingdom) at 4°C. And then the sections were washed with PBS and incubated with gout anti-rabbit secondary antibody at 37°C for 30 min. Finally, the tissue sections were washed with PBS, and visualized by using the protocol supplied by the manufacturer.

The levels of immunohistochemical was testified by staining German immunoreactive score (IRS). The percent positivity was scored as “0” (<5%), “1” (5%–25%), “2” (26%–50%), “3” (51%–75%) and “4” (>75%). The staining intensity was scored as “0” (negative), “1” (weak), “2” (moderate) and “3” (strong). And then they were multiplied to yield the IRS score, ranging from 0 to 12. Scores were considered negative (0–1), weakly positive (2–4), moderately positive (6–8) and strongly positive (9–12).

### Statistical Analyses

All the statistical analyses were performed with R software (version 4.0.2; http://www.Rproject.org). Univariate Cox regression analysis, random survival forest algorithm, and multivariate Cox regression were used to construct the risk score model by using the “survival” and “randomForestSRC” packages. The time-dependent receiver operating characteristic (ROC) analysis and Kaplan-Meier survival analysis were chosen to test the prognostic value of the signature *via* the “survivalROC” and “survminer” packages. The KEGG and GO pathway enrichment analysis was performed by “clusterProfiler” package. The nomogram was generated by the “rms” package. *p*-value < 0.05 was considered as significant unless otherwise specified.

## Results

### Selection and Validation of Hub Genes

The hypoxia-related gene set downloaded from the GSEA database contained 200 genes up-regulated in response to a low oxygen level. A total of 48 genes were selected by univariate Cox regression analysis as they were significantly associated with bladder cancer patients’ DSS. Then the random survival forest algorithm was conducted for variables hunting and eight genes were identified to be core genes (Supplementary Table S2, Supplementary Figure S1). After further screening through the multiple Cox regression analysis, four hypoxia-related genes (ANXA2, GALK1, COL5A1, and HS3ST1) were selected to construct the prognostic model (Table 1), and the Kaplan-Meier survival curves of these four genes were provided in Supplementary Figure S1.

**TABLE 1:** Cox regression analysis of genes associated with disease-specific survival of bladder cancer patients.

Variables	Univariate cox regression		Multiple cox regression			
	HR^a^	p-value	Coef	HR	p-value	95% CI^b^ of HR
ANXA2	3.56	<0.01	0.78	2.17	0.02	1.14–4.14
HS3ST1	0.52	<0.01	-0.31	0.73	0.12	0.50–1.09
COL5A1	1.73	<0.01	0.20	1.22	0.13	0.94–1.57
GALK1	3.82	<0.01	0.76	2.15	0.07	1.04–4.44

a HR, hazard ratio.

b CI, confidence interval.

**FIGURE 2:**
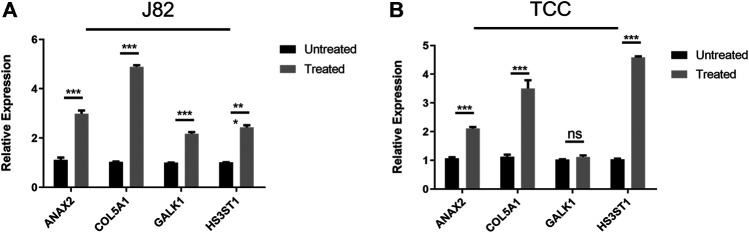
Validation of the expression of hub genes after culturation in hypoxia environment **(A)** J82 cell line **(B)** TCC cell line.

Besides, quantitative real-time PCR was performed to validate the changes of the expression of these four genes in the hypoxia environment. The results revealed that all these four genes upregulated after culturation in 1% O_2_ for 24 h (Figure 2), which were consistent with previous studies.

### Construction and Validation of the Risk Signature

According to the results of the multiple Cox regression analysis, the risk score for each patient was calculated as follows:

Risk score = (0.7752 × expression of ANXA2) + (0.7640 × expression of GALK1)+ (0.1973 × expression of COL5A1) +  (− 0.3086 × expression of HS3ST1)

**FIGURE 3:**
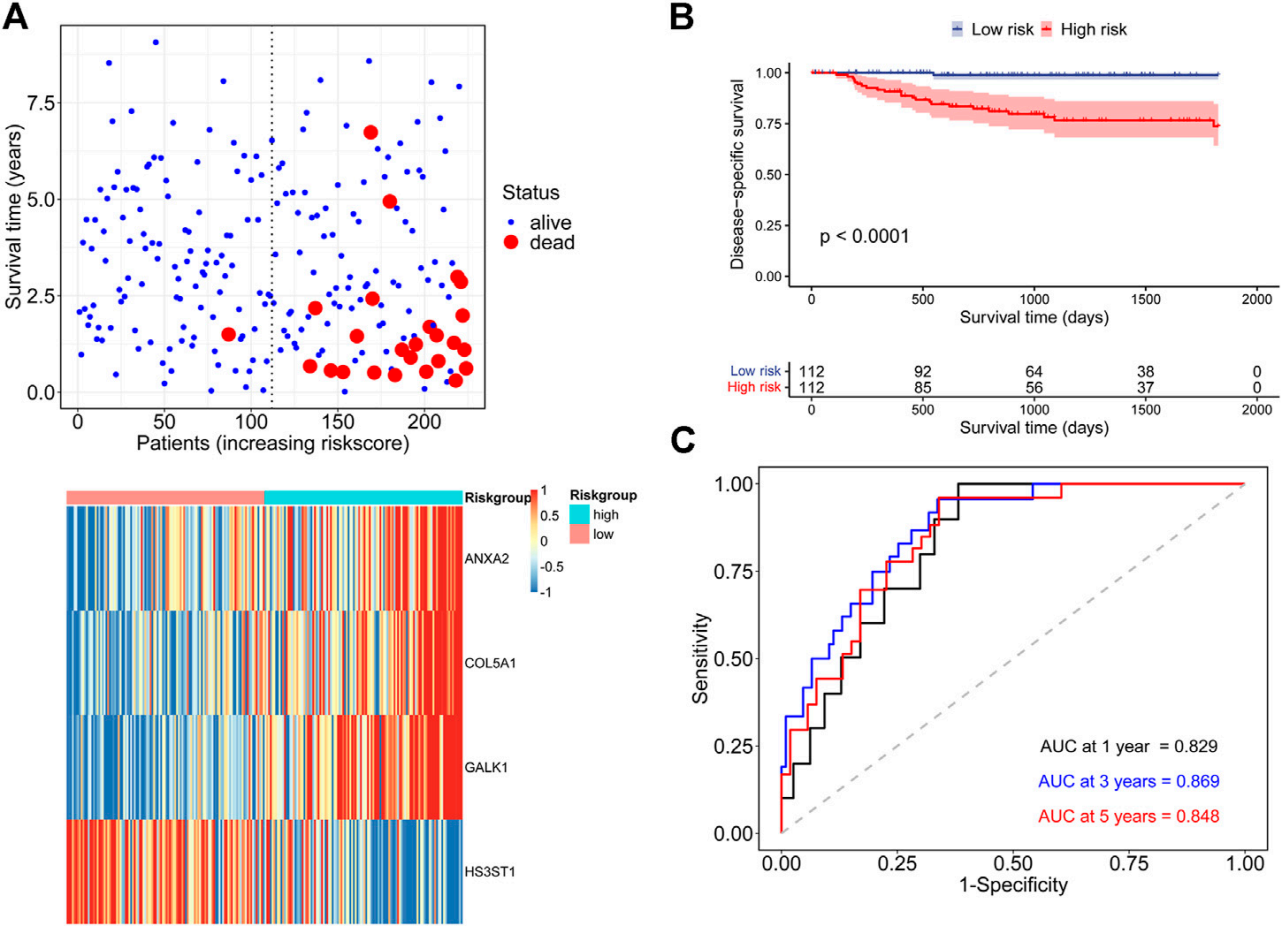
Assessment of the prognostic ability of the hypoxia-related signature in the discovery cohort **(A)** DSS status and core genes expression heatmap in two risk groups **(B)** Kaplan-Meier survival analysis according to the signature **(C)** Time-dependent ROC analysis for DSS prediction.

Then patients in the discovery cohort were equally divided into low-risk and high-risk groups based on the median risk score. The distribution of survival status and the hub genes expression heatmap for each patient were exhibited in Figure 3A. The Kaplan-Meier survival analysis indicated that patients in high-risk group had a higher DSS rate than those in low-risk group (Figure 3B). The time-dependent ROC analysis showed that the signature exhibited an excellent ability to predict the DSS rate and the areas under the curve (AUC) of the hypoxia-related signature were 0.829 at 1 year, 0.869 at 3 years, and 0.848 at 5 years, respectively (Figure 3C). Besides, the concordance index (C-index) of the signature was 0.839, representing a high prediction accuracy.

**FIGURE 4:**
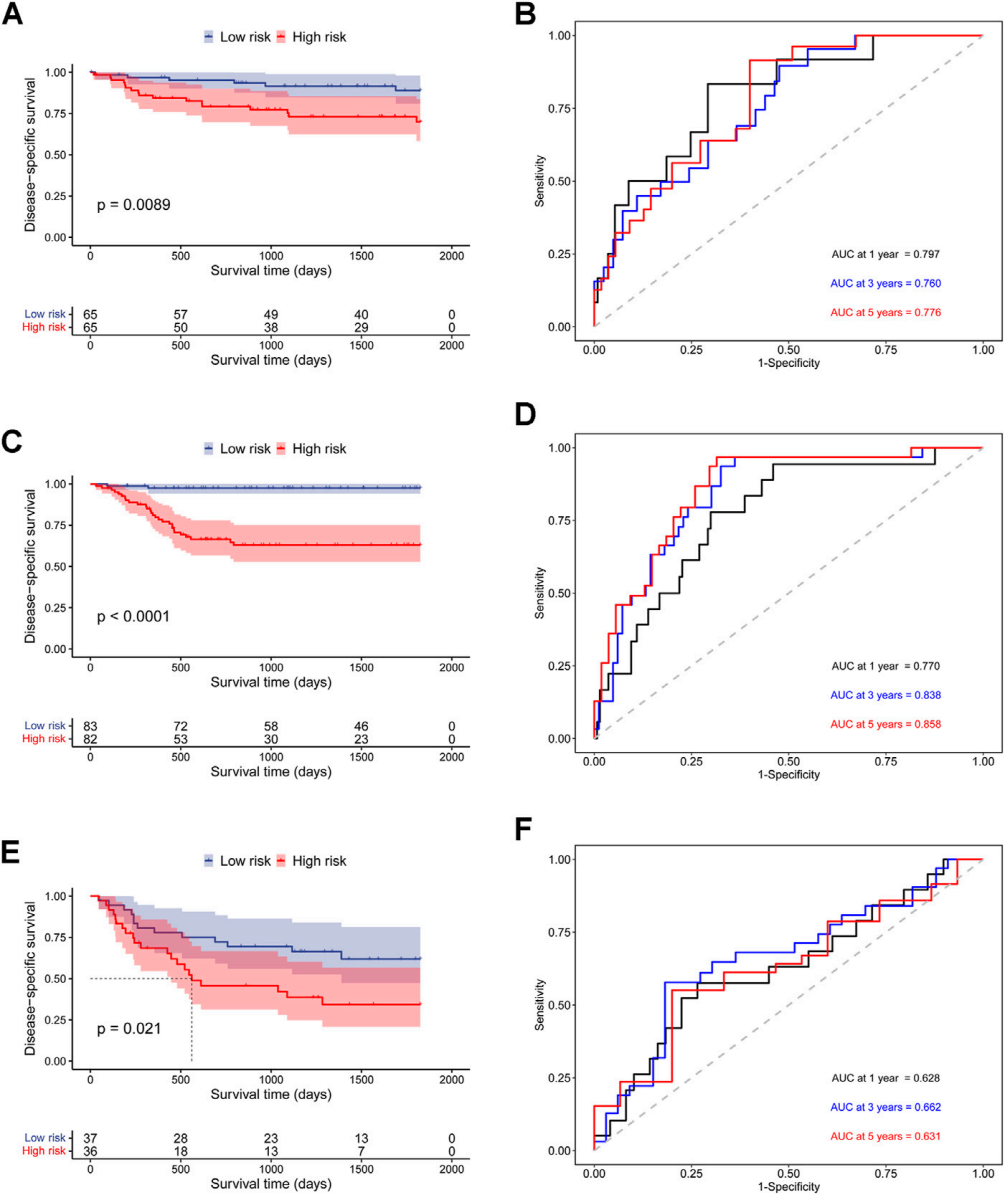
Validation of the prognostic ability of the hypoxia-related signature for DSS prediction in validation cohorts. **(A)** Kaplan-Meier curves for patients in GSE32548 cohort; **(B)** Time-dependent ROC curves for patients in GSE32548 cohort; **(C)** Kaplan-Meier curves for patients in GSE13507 cohort; **(D)** Time-dependent ROC curves for patients in GSE13507 cohort; **(E)** Kaplan-Meier curves for patients in GSE48075 cohort; **(F)** Time-dependent ROC curves for patients in GSE48075 cohort.

**FIGURE 5:**
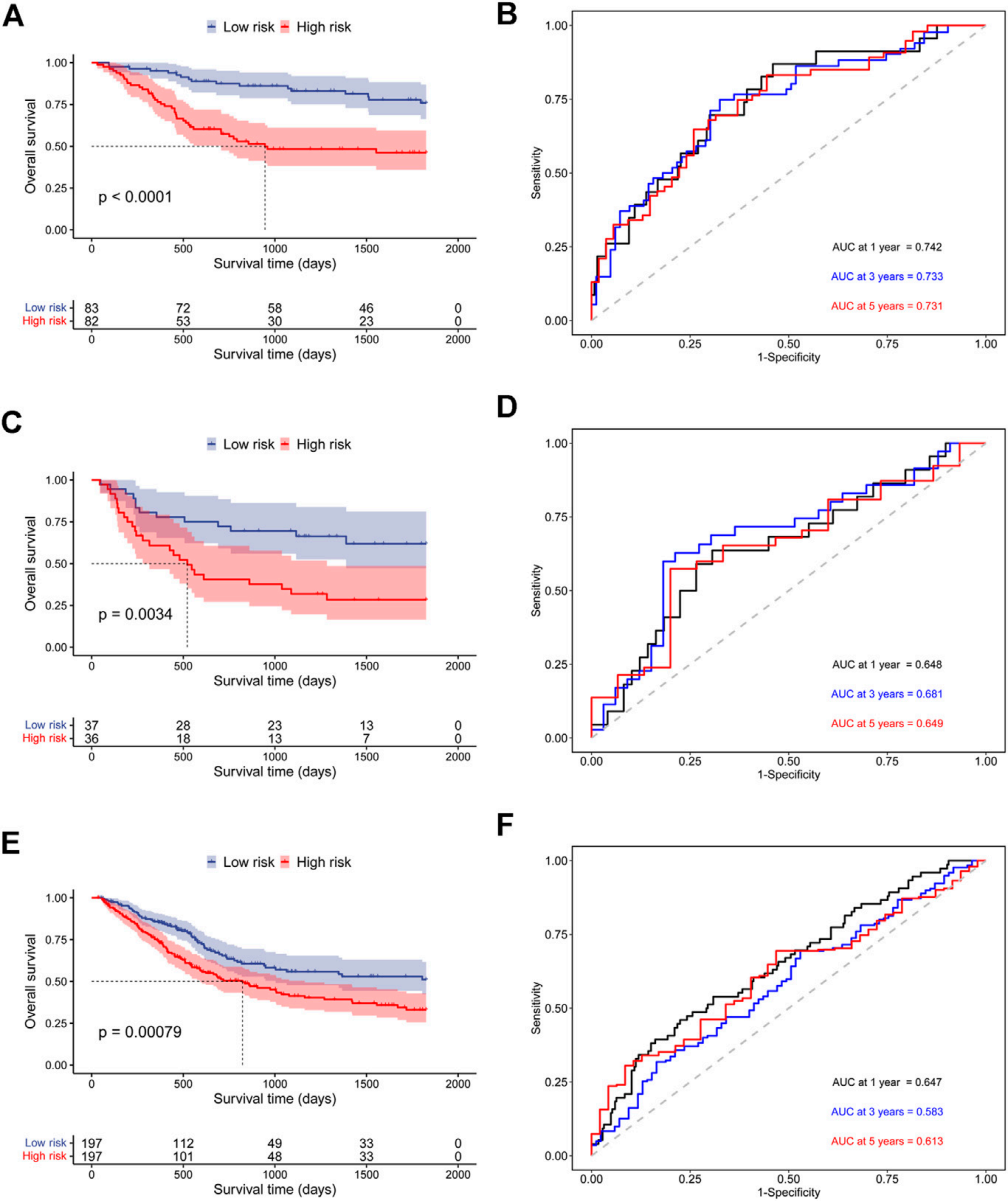
Validation of the prognostic ability of the hypoxia-related signature for OS prediction in validation cohorts. **(A)** Kaplan-Meier curves for patients in GSE13507 cohort; **(B)** Time-dependent ROC curves for patients in GSE13507 cohort; **(C)** Kaplan-Meier curves for patients in GSE48075 cohort; **(D)** Time-dependent ROC curves for patients in GSE48075 cohort; **(E)** Kaplan-Meier curves for patients in TCGA-BLCA cohort; **(F)** Time-dependent ROC curves for patients in TCGA-BLCA cohort.

In addition, to further prove the prediction value of this signature, three external cohorts (GSE13507, GSE32548, and GSE48075) were used for DSS validations and three external cohorts (GSE13507, GSE48075, TCGA-BLCA) were used for OS validations. Surprisingly, the results demonstrated that patients in high-risk group had a poorer prognosis than those in low-risk group regardless of DSS (Figure 4) and OS (Figure 5), which were consistent with the results in the discovery cohort. Moreover, the time-dependent ROC analysis showed that this signature had a high accuracy in all validation cohorts. In conclusion, this hypoxia-related signature has certain reliability and might be an effective candidate for the prediction of bladder cancer patients’ survival status.

### Correlation Between the Hypoxia-Related Signature and Clinical Characteristics

**FIGURE 6:**
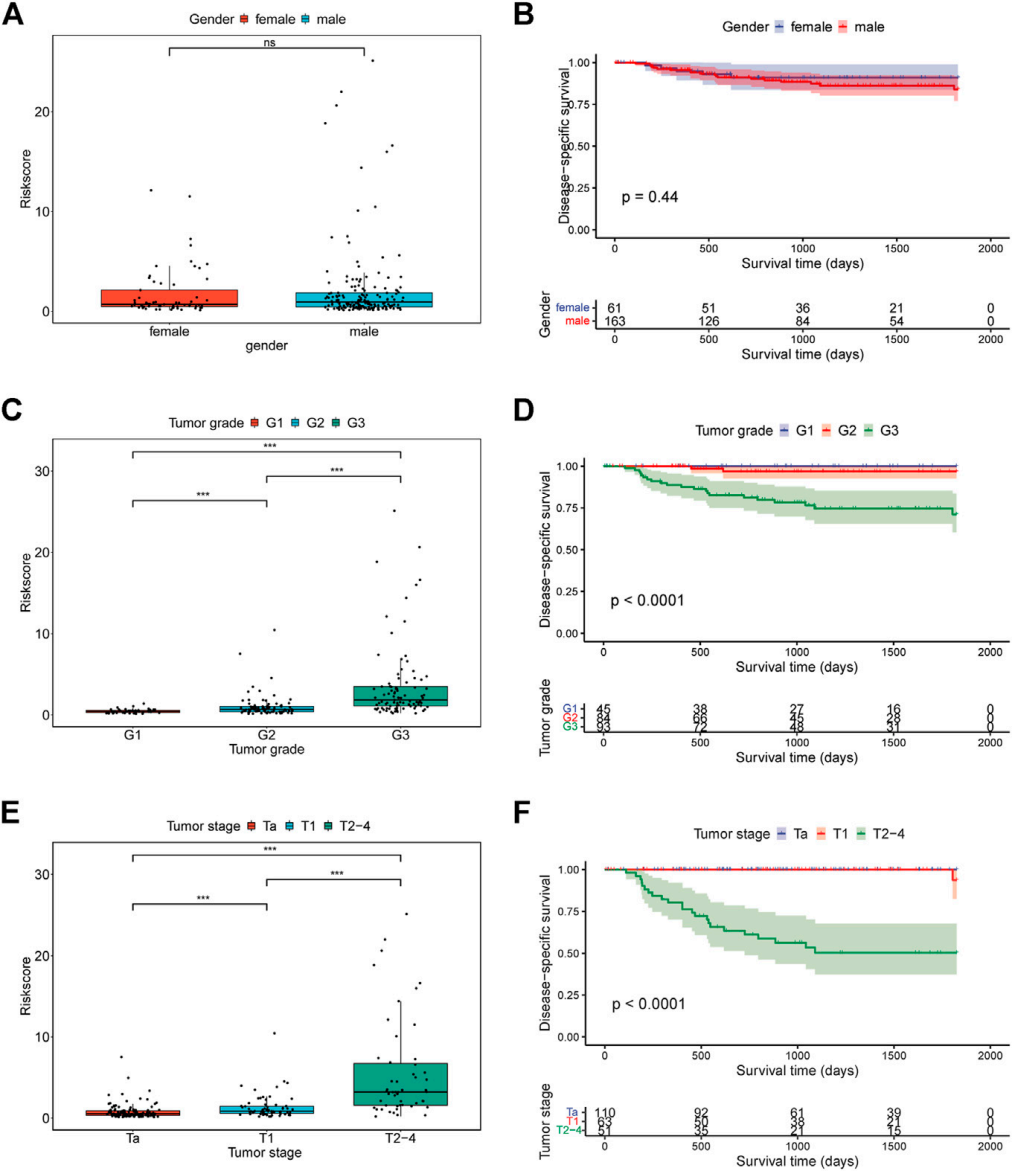
The relationship between risk score and clinical characteristics. **(A)** The relationship between risk score and gender; **(B)** Kaplan-Meier curves for patients in GSE32894 cohort based on gender; **(C)** The relationship between risk score and tumor grade; **(D)** Kaplan-Meier curves for patients in GSE32894 cohort based on tumor grade; **(E)** The relationship between risk score and tumor stage; **(F)** Kaplan-Meier curves for patients in GSE32894 cohort based on tumor stage.

To investigate the association between clinical characteristics and our signature, patients were stratified into different subgroups base on gender, tumor grade, tumor stage, respectively. Surprisingly, we found the risk scores of patients were significantly different among tumor grade subgroups and tumor stage subgroups, which were closely related with patients’ DSS (Figures 6B,C). However, no significance of the risk scores was observed between female patients and male patients (Figure 6A). In addition, the Kaplan-Meier survival analysis for patients in different subgroups revealed that the risk signature could efficiently stratify patients into different risk groups in most of the subgroups (Fig. S3).

### Establishment and Assessment of the Nomogram

**FIGURE 7:**
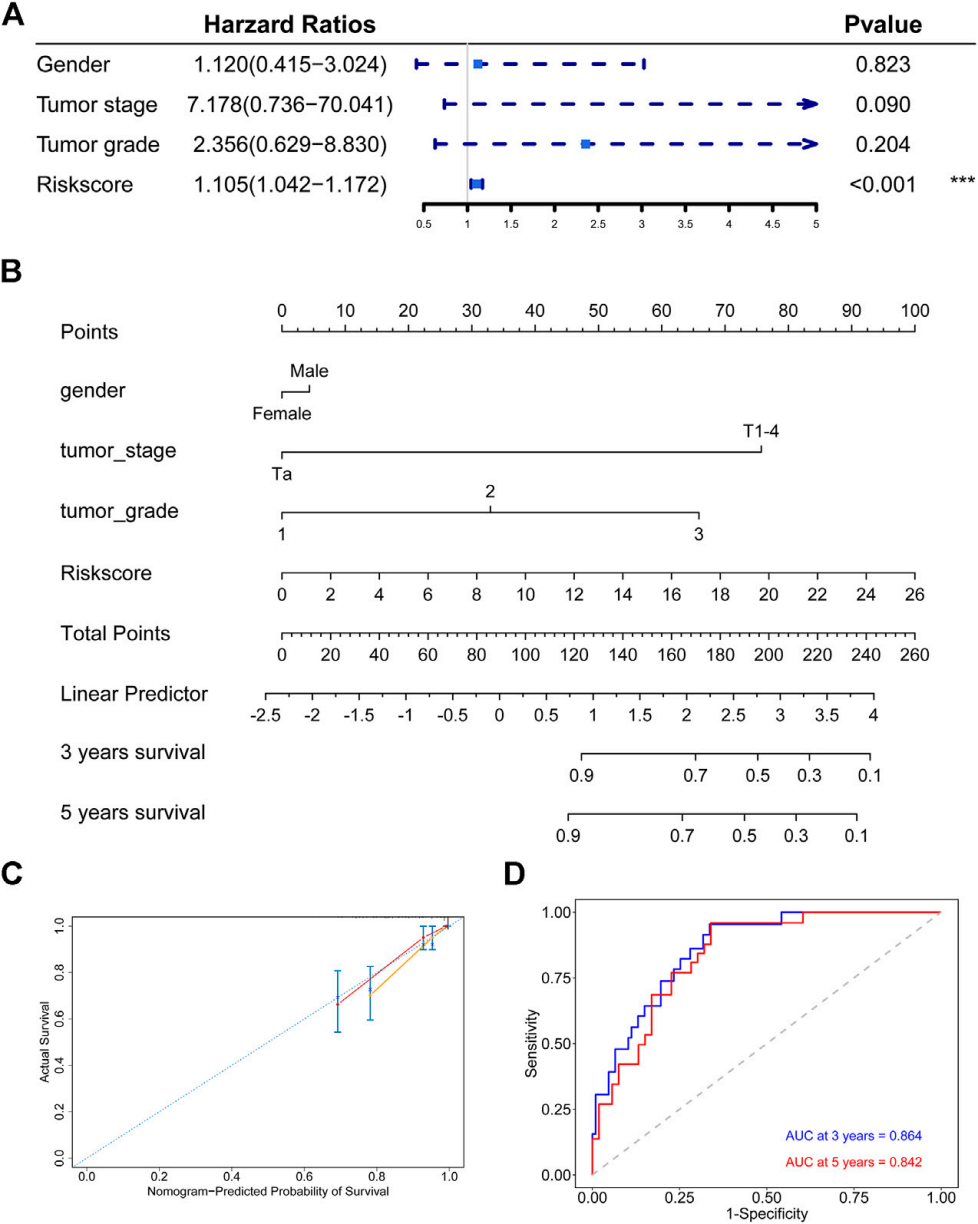
Establishment and assessment of the nomogram in the discovery cohort **(A)** The signature is the only independent fact in GSE32894 cohort **(B)** Nomogram based the signature and clinical characteristics **(C)** The 3-years and 5-years calibration plots for the nomogram **(D)** Time-dependent ROC analysis for the nomogram.

We performed a multivariate Cox regression analysis to assess the prognostic value of the hypoxia-related signature and other clinical features. The results revealed that our risk signature was the only independent prognostic factor in the GSE32894 cohort (Figure 7A) and an independent prognostic factor in most of other cohorts (Supplementary Figure S4). Besides, we also compared the prognosis value of the signature with other known prognosis related factors. Our signature was found to have a larger AUC than other risk factors, indicating a high prognosis prediction value for bladder cancer patients (Supplementary Table S6).

We constructed a nomogram to predict the 3-years and 5-years DSS probabilities of each bladder cancer patient, which combined our hypoxia-related signature and other clinical characteristics (Figure 7B). Each patient could receive a score and a DSS rate by this nomogram. The 3-years and 5-years calibration plots indicated that the nomogram exhibited good agreement to the actual survival status (Figure 7C). Besides, the time-dependent ROC analysis showed that our nomogram had a large AUC and the C-index of the nomogram was 0.846, which meant that the nomogram had a good prediction ability (Figure 7D).

### Gene Set Enrichment Analysis Between Different Risk Groups

**FIGURE 8:**
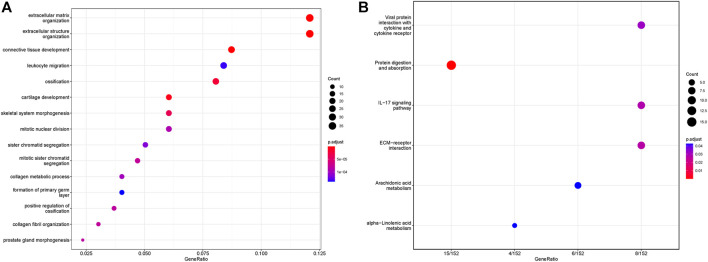
GSEA analysis between two risk groups **(A)** GO analysis **(B)** KEGG analysis.

We performed Gene Set Enrichment Analysis (GSEA) between the high-risk group and low-risk group to identify the underlying mechanism. GO and KEGG pathway enrichment analyses were operated and the results were shown in Figure 8. In the GO analysis, extracellular matrix organization, extracellular structure organization, connective tissue development, leukocyte migration were the top five enrichment pathways. And in the KEGG analysis, we found our signature was associated with viral protein interaction with cytokine and cytokine receptor, protein digestion and absorption, IL-17 signaling pathway, ECM-receptor interaction, arachidonic acid metabolism, and alpha-linolenic acid metabolism.

### The Signature was a Predictive Biomarker for the Immune Microenvironment

**FIGURE 9:**
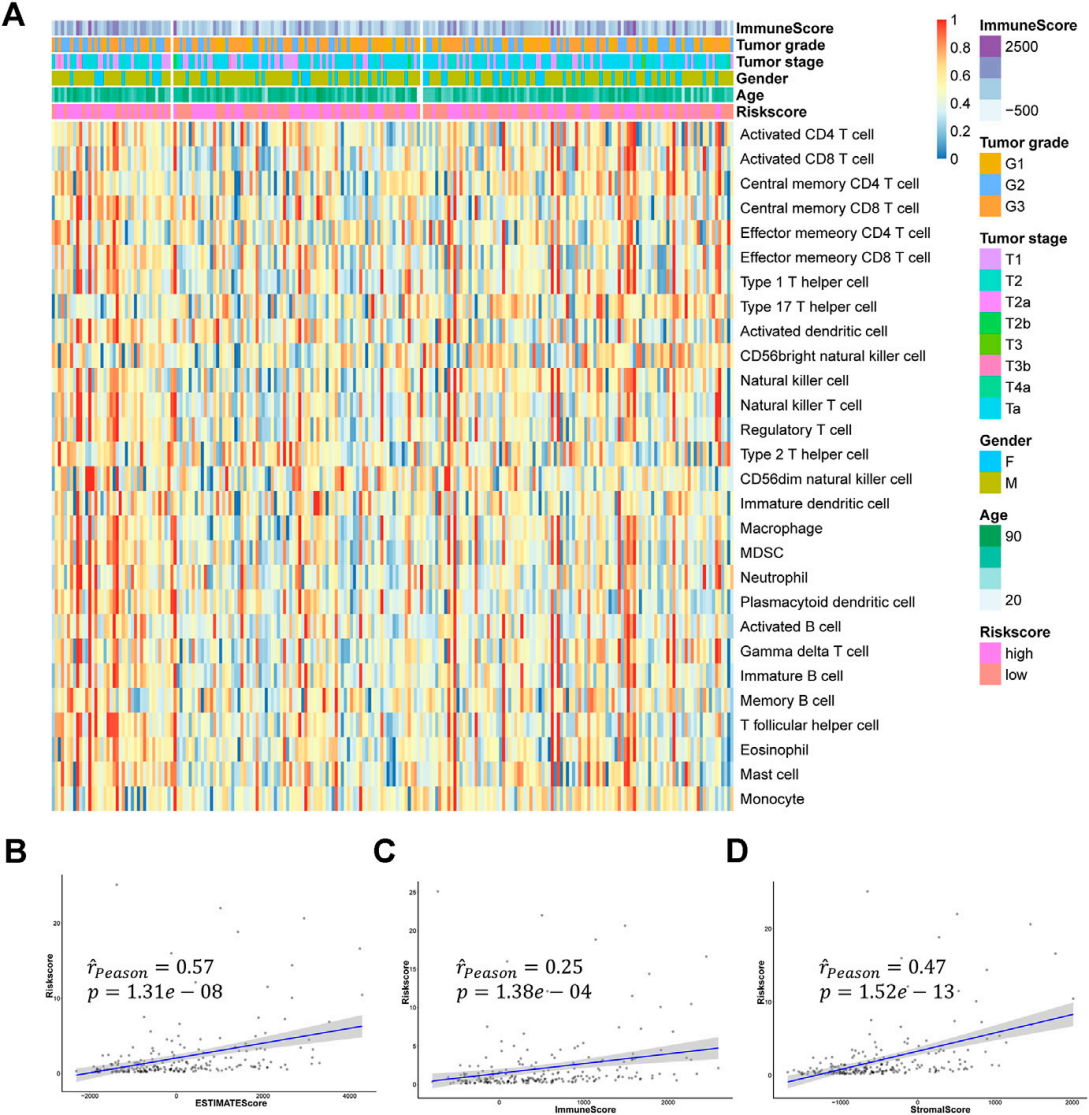
The relationship between hypoxia-related signature and TME **(A)** The heatmap of 28 immune cells infiltration **(B–D)** The relationships between the risk score and ESTIMATEScore, ImmuneScore, and StromalScore.

To explore the relation of the signature to the IME, we estimated the relative abundance of 28 immune cells in the GSE32894 cohort by the ssGSEA algorithm. As shown in the Figure 9A and Supplementary Figure S5, we found most of the 28 immune cells were highly infiltrated in the high-risk group. We also calculated the ImmuneScore, StromalScore, and ESTIMATEScore for each patient and found a strong correlation between risk score and these immune-related scores (Figures 9B–D).

**FIGURE 10:**
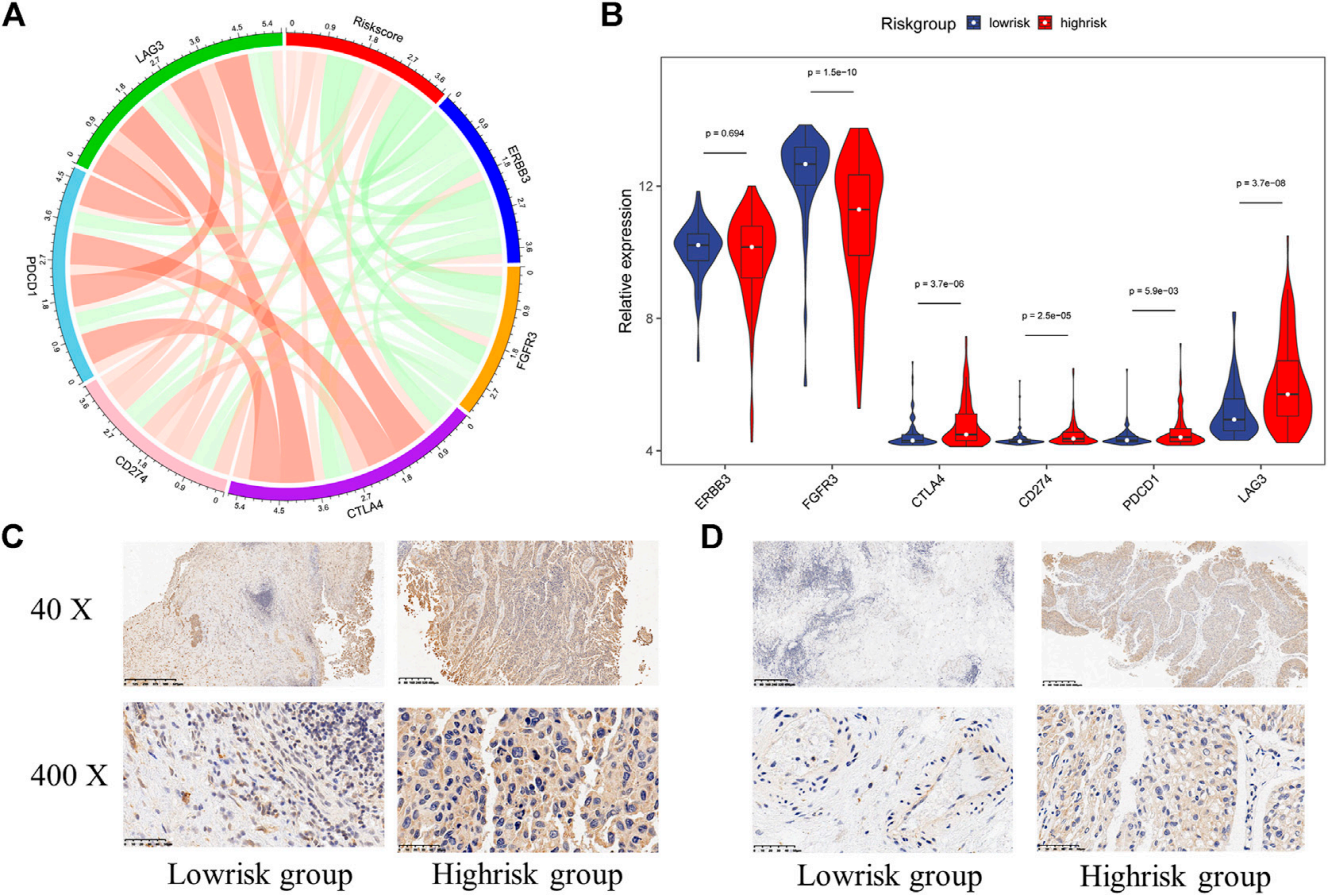
The relationship between hypoxia-related signature and immune checkpoints **(A)** The chord diagram used to visualize the relationship between risk score and immune checkpoints expression **(B)** The expression characteristics of immune checkpoints in different risk groups **(C)** Immunohistochemistry detection of CTLA4 in bladder cancer tissues **(D)** Immunohistochemistry detection of LAG3 in bladder cancer tissues.

As previous researches reported, immunotherapy has shown great potential in bladder cancer. The expression of immune checkpoints was regarded to be highly correlated with the response to immunotherapy. Therefore, we assessed the relationships between the signature and some important immune checkpoints, including ERBB3, FGFR3, CTLA4, CD274 (PD-L1), PDCD1 (PD-1), and LAG3. Surprisingly, almost all checkpoints except ERBB3 significantly differentially expressed between the high-risk group and low-risk group. And we could also find FGFR3 was negatively correlated with the risk score while CTLA4, CD274, PDCD1, and LAG3 were positively correlated with the risk score (Figures 10A,B). Furthermore, we performed IHC experiments on 14 bladder cancer samples. The bladder cancer samples were divided into high risk group and low risk group based on the median risk score, and the results of IHC indicated the positive frequencies of CTLA4 and LAG3 were higher in patients in high risk group (Figures 9C,D, Supplementary Table S9). Above all, our signature had the potential to be a promising biomarker for predicting the efficacy of immunotherapy.

## Discussion

Bladder cancer is one of the most frequent cancers in the world, and it is a great threat to human health. Unlike many other cancers, the diagnostics, treatments, and five-year survival rates for bladder cancer are largely unchanged since the 1990s. Clinicians usually use clinical characteristics like tumor grade, tumor stage, and age, to predict the progression and prognosis of bladder cancer patients. However, these characteristics provide only limited information and may be unreliable in most cases (Gospodarowicz, 1994), as bladder cancer is a highly heterogeneous disease. With the development of genome sequencing, some novel molecular biomarkers have been screening out to predict the prognosis and offer personal treatment guidance for reference (Dyrskjot et al., 2017).

Hypoxia is a characteristic hallmark of the TME. Previous researches have revealed that hypoxia can promote cancer progression and metastasis. In this study, we selected four hypoxia-related genes that were highly related to bladder cancer patients’ survival status, including ANXA2, GALK1, COL5A1, and HS3ST1, to construct a signature and this signature is independent of other clinical characteristics. Among these genes, ANXA2 belongs to the Annexin family and is reported to work in several tumor cellular processes, such as cell cycle regulation (Chiang et al., 1993), angiogenesis (Ling et al., 2004), tumor invasion, and progression (Sharma et al., 2006). ANXA2 has been regarded as a biomarker in bladder cancer, clear cell renal cell carcinoma, hepatocellular carcinoma, and prostate cancer (Christensen et al., 2018). Accumulating researches indicated that COL5A1 might be a new prognostic candidate for many carcinomas (Chen et al., 2019; Cao et al., 2020). Besides, COL5A1 can accelerate the growth and progression of gastric cancer (Wei et al., 2020). HS3ST1 is a member of the heparan sulfate biosynthetic enzyme family and has been reported to be associated with the NF-kB signaling pathway in acute lymphocytic leukemia (Zhang et al., 2019). GALK1 is reported to be a novel candidate therapeutic target for hepatocellular carcinoma as its important role in protein glycosylation (Tang et al., 2016). No research on the relationship between GALK1 and bladder cancer has been found yet.

The correlation between hypoxia feature and IME has been previously reported in many cancers. Stéphane Terry found hypoxia might play a central role in the regulation of intratumor heterogeneity and immune evasion (Terry et al., 2020). Giovarelli has shown that hypoxia has effects on differentiation, adaptation, and activation of dendritic cells in tumors (Elia et al., 2008). Hypoxia can also downregulate the expression and function of most NK cell receptors that are directly responsible for exerting cytolytic activity against tumor cells (Balsamo et al., 2013a). In our research, we found the hypoxia-related signature was highly relevant to the immune cell infiltration levels.

In the past few years, immunotherapy has a prominent place in the treatment for bladder cancer. Unlike the conventional treatments, such as surgery, chemotherapy, and radiotherapy, immunotherapy may provide an individualized and effective choice for each bladder cancer patient. Bacillus Calmette–Guerin (BCG) is the first immunotherapy drug approved by Food and Drug Administration (FDA) for bladder cancer and has become a gold standard for high-risk NMIBC. However, about 25–45% of bladder cancer patients would not response to BCG therapy and some severe adverse reactions have been found in patients treated by BCG (Pettenati and Ingersoll, 2018; Crispen and Kusmartsev, 2020; Han et al., 2020). As a supplement, several immune checkpoint inhibitors (ICIs) have been approved for the treatment of bladder cancer (Popovic et al., 2017). Multiple clinical trials have demonstrated that survival rate and side effect rate may remarkably improve after receiving ICIs, such as PD-L1 inhibitors and PD-1 inhibitors (Zhou et al., 2017; Galsky et al., 2018; Powles et al., 2018). Besides, ERBB3, FGFR3, CTLA4, and LAG3 are also reported in some studies as potential therapeutic targets for bladder cancer (Tomlinson et al., 2007; Cancer Genome Atlas Research, 2014; Gofrit et al., 2014; Molyneaux et al., 2015; Vidotto et al., 2019). Unfortunately, only few patients can benefit from ICIs therapy. Therefore, it is of great importance to develop a signature for predicting the efficacy of ICIs therapy. In this study, we found the hypoxia-related signature was highly corelated to most of immune checkpoints, indicating it may be a promising biomarker for the efficacy of ICIs therapy.

In general, we constructed a hypoxia-related signature which has exhibited great potential in predicting the survival status and the efficacy of immunotherapy. However, there were still some certain limitations to this research. First, as this research was retrospective, the results were biased to an extent. Second, the clinical information from our cohorts was not complete, which might affect the prediction accuracy of our nomogram. Furthermore, our sample size was limited, the signature should be verified in multicenter clinical trials with more bladder cancer patients.

## Conclusion

In conclusion, we constructed and validated a hypoxia-related prognostic signature that could effectively classify bladder cancer patients into high and low-risk groups. Besides, this signature had a good ability to characterize the IME feature and predict the efficacy of immunotherapy. These findings may provide useful and individual guidance in clinical work.

## Data Availability

Publicly available datasets were analyzed in this study. This data can be found here: Gene Expression Omnibus (https://www.ncbi.nlm.nih.gov/geo/) database and The Cancer Genome Atlas (https://portal.gdc.cancer.gov/).
