# Supplementary material for: Identification of a Hypoxia-Related Signature for Predicting Prognosis and the Immune Microenvironment in Bladder Cancer
Source: Front Mol Biosci. 2021 May 7;8:613359. doi: 10.3389/fmolb.2021.613359 (PMC8138130; doi:10.3389/fmolb.2021.613359)
Supplement: Supplementary file 1 [file datasheet1.docx]

Supplementary Material

# Supplementary Figures and Tables

## Supplementary Figures

**
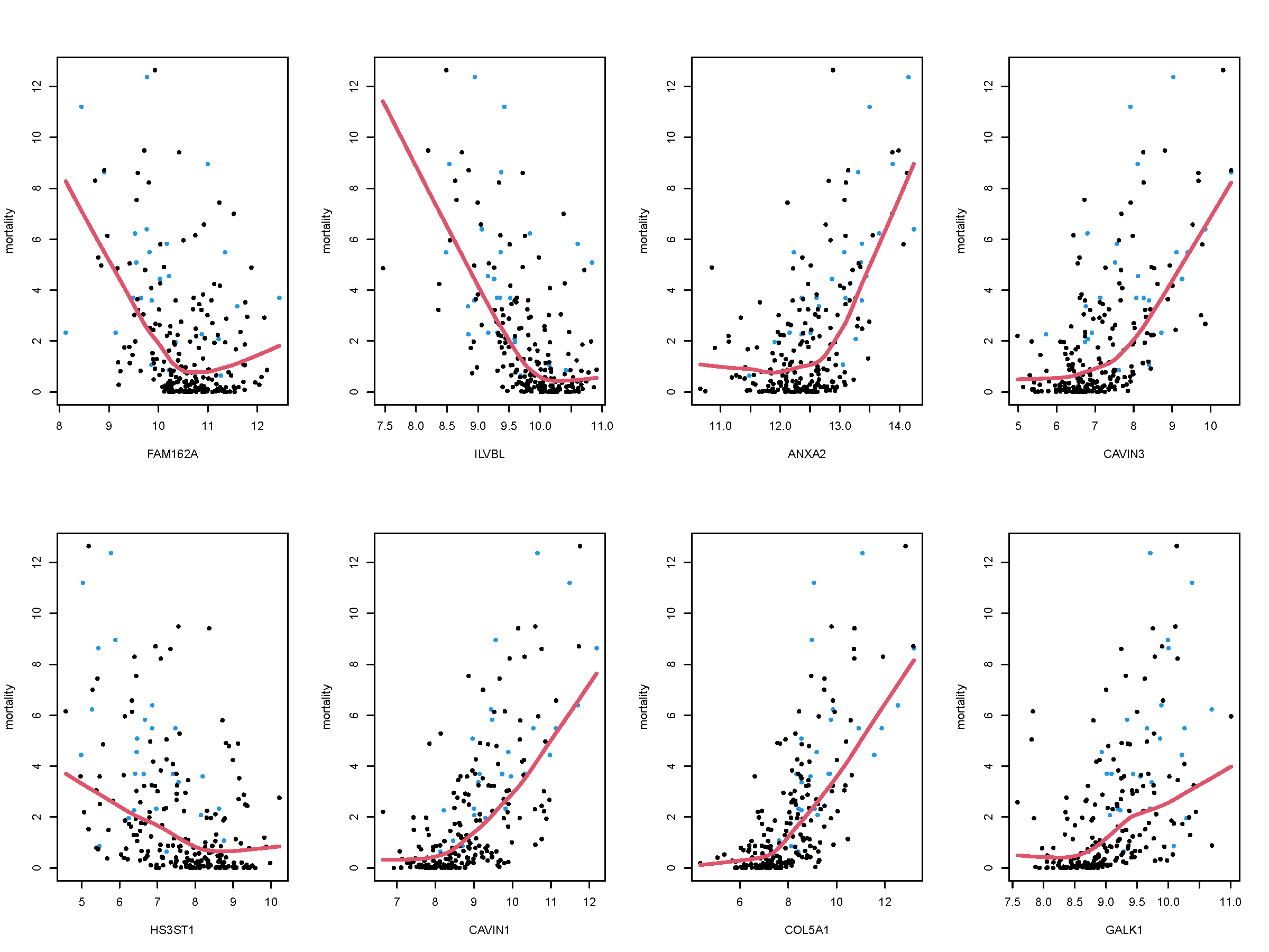
**

**Figure S1.** Visualization of variable hunting analysis for identifying the core genes.

**
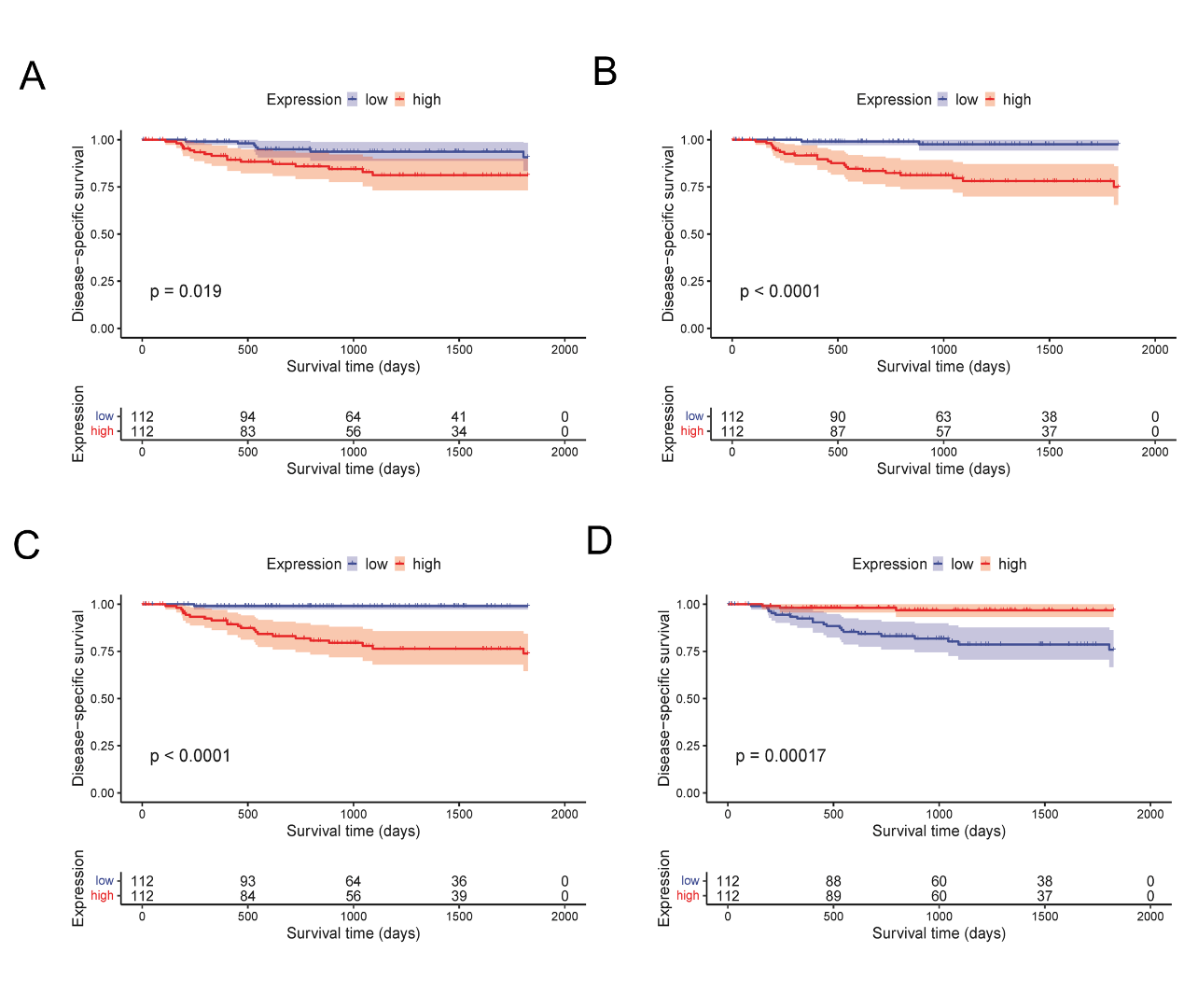
**

**Figure S2.** Kaplan–Meier survival analysis of four hypoxia-related genes. (A) ANXA2; (B) COL5A1; (C) GALK1; (D) HS3ST1.


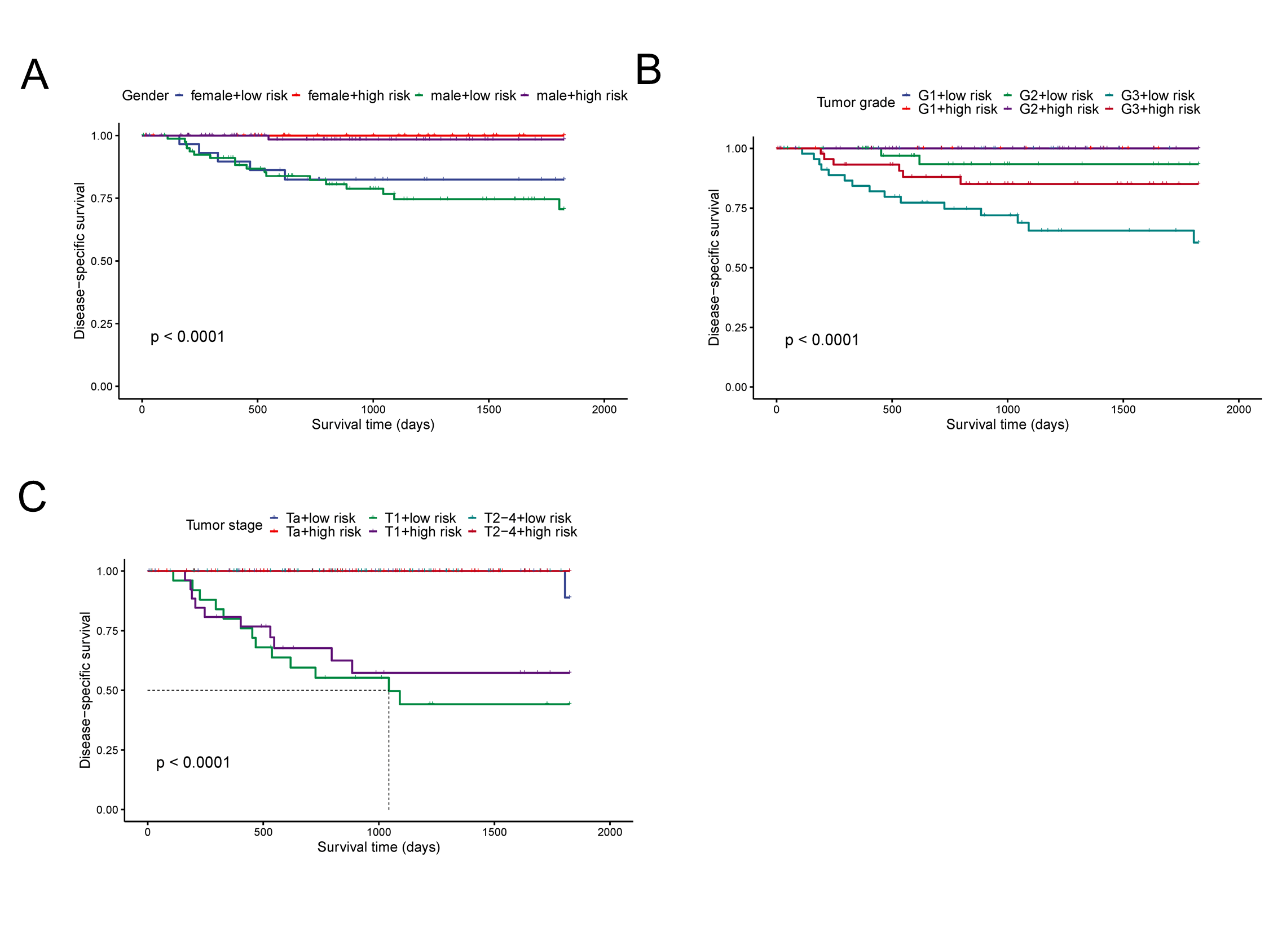


**Figure S3.** Kaplan–Meier survival analysis for patients in different subgroups according to the signature. (A) Patients were stratified by gender (female or male); (B) Patients were stratified by tumor grade (G1, G2, or G3); (C) Patients were stratified by tumor stage (Ta, T1, or T2-4).


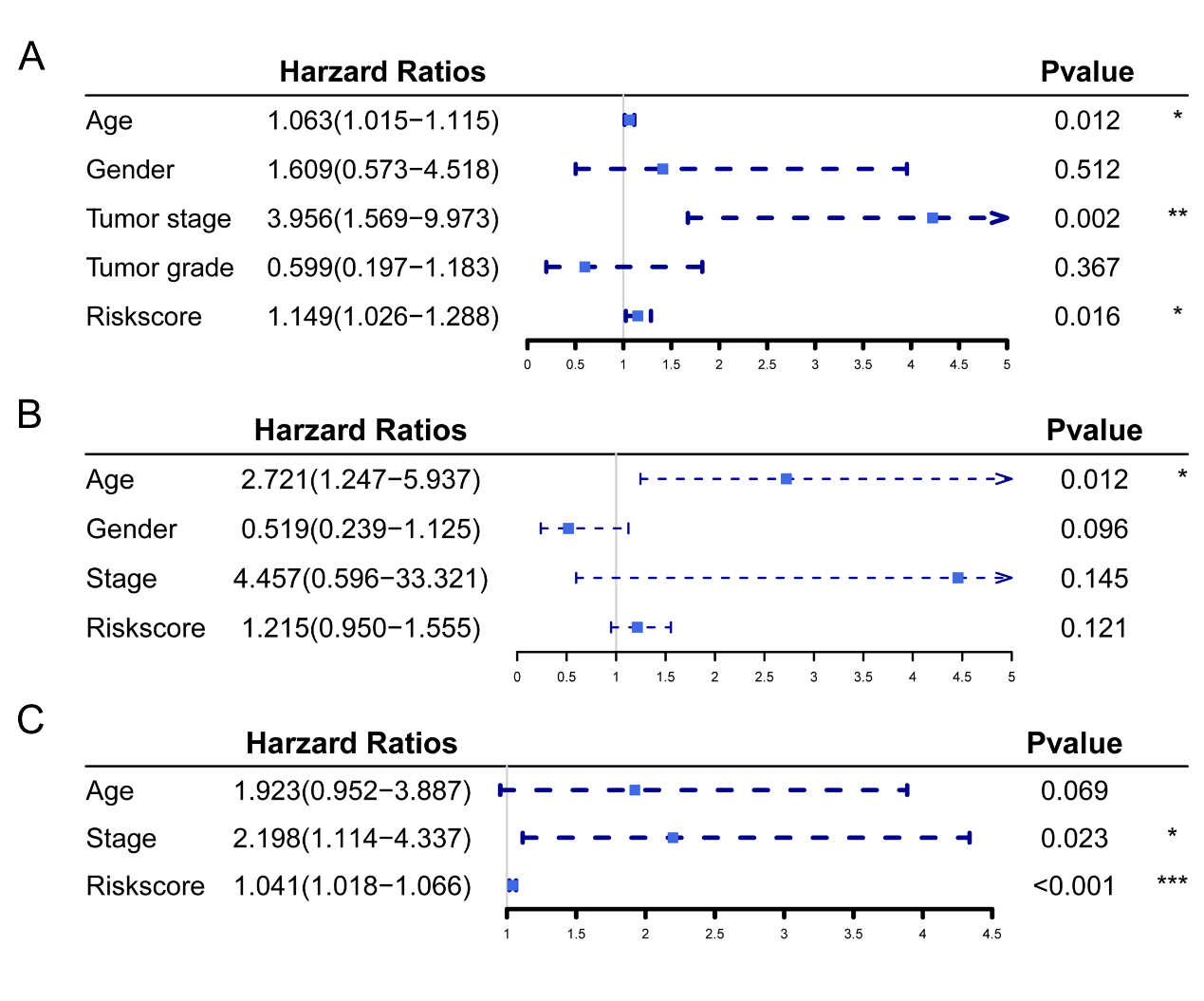


**Figure S4.** Multiple Cox regression analysis of risk score and clinical characteristics in validation cohorts. (A) GSE32548; (B) GSE13507; (C) GSE48075.


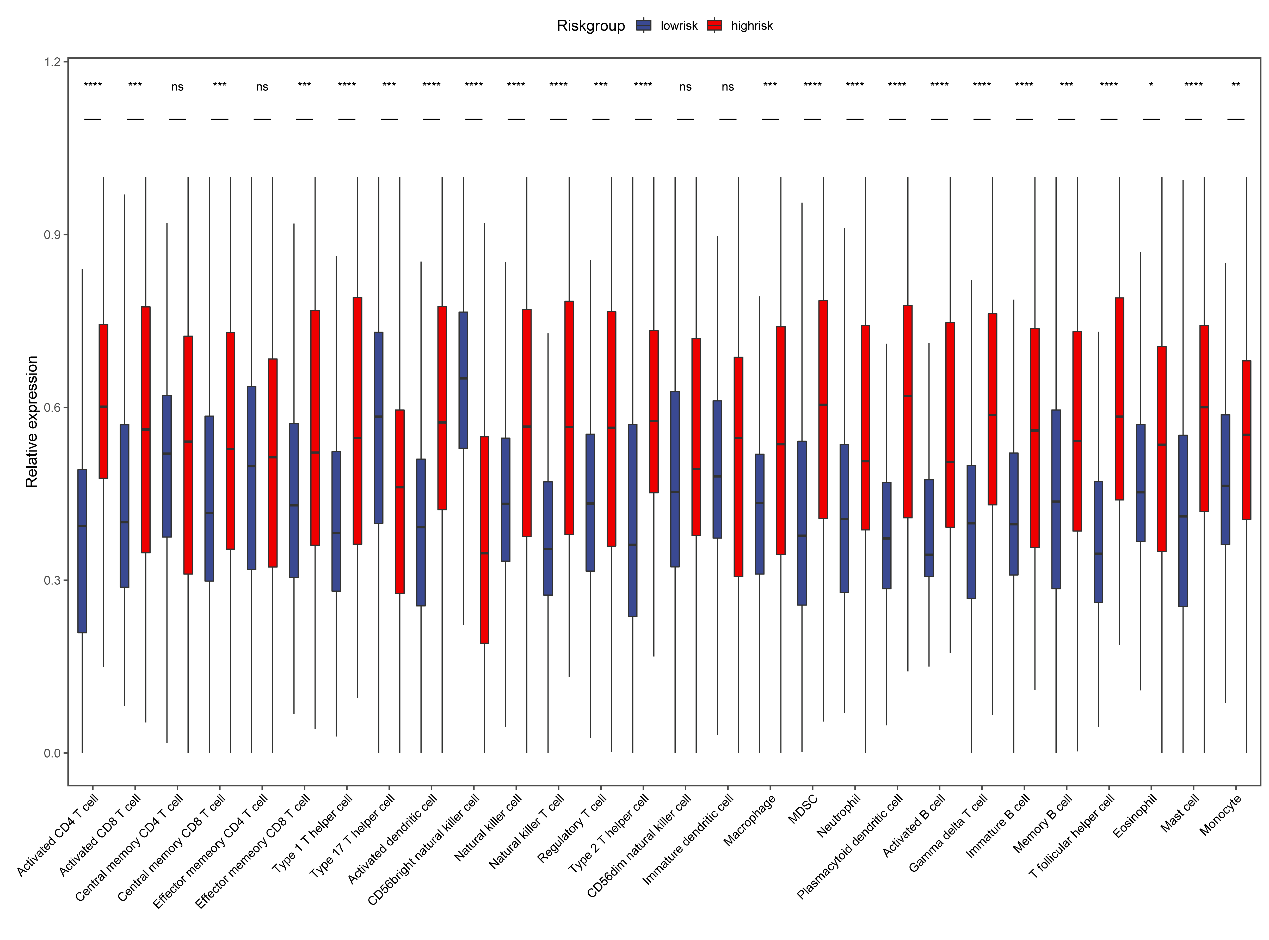


**Figure S5.** The relationship between risk score and the relative abundance of 28 immune cells.

## Supplementary Tables

**Table S1. Clinical characteristics in GSE32894 cohort**

| **Characteristics** | **Number of patients (percent)** |
| --- | --- |
| **Gender** |  |
| Male | 163 (72.8%) |
| Female | 61 (27.2%) |
| **Grade** |  |
| Gx | 2 (0.9%) |
| G1 | 45 (20.1%) |
| G2 | 84 (37.5%) |
| G3 | 93 (41.5%) |
| **Pathology_T_stage** |  |
| Ta | 110 (49.1%) |
| T1 | 63 (28.1%) |
| T2 | 43 (19.2%) |
| T3 | 7 (3.1%) |
| T4 | 1 (0.4%) |
| **Disease-specific survival status** |  |
| Alive | 199 (88.8%) |
| Dead | 25 (11.2%) |
| **Total** | 224 (100%) |

**Table S2. Clinical characteristics in the GSE32548 cohort**

| **Characteristics** | **Number of patients (percent)** |
| --- | --- |
| **Gender** |  |
| Male | 99 (76.2%) |
| Female | 31 (23.8%) |
| **Age** |  |
| <=70 | 65 (50.0%) |
| >70 | 65 (50.0%) |
| **Tumor grade** |  |
| G1 | 15 (11.5%) |
| G2 | 40 (30.8%) |
| G3 | 75 (57.7%) |
| **Tumor stage** |  |
| Tx | 1 (0.8%) |
| Ta | 40 (30.8%) |
| T1 | 51 (39.2%) |
| ≥T2 | 38 (29.2%) |
| **Disease-specific survival status** |  |
| Alive | 105 (80.8%) |
| Dead | 25 (19.2%) |
| **Total** | 130 (100%) |

**Table S3. Clinical characteristics in the GSE13507 cohort**

| **Characteristics** | **Number of patients (percent)** |
| --- | --- |
| **Gender** |  |
| Male | 135 (81.8%) |
| Female | 30 (18.2%) |
| **Age** |  |
| <=70 | 109 (66.1%) |
| >70 | 56 (33.9%) |
| **Subtypes** |  |
| MIBC | 62 (37.6%) |
| NMIBC | 103 (62.4%) |
| **Disease-specific survival status** |  |
| Alive | 133 (80.8%) |
| Dead | 32 (19.2%) |
| **Overall survival status** |  |
| Alive | 96 (58.2%) |
| Dead | 69 (41.8%) |
| **Total** | 165 (100%) |

**Table S4. Clinical characteristics in the GSE48075 cohort**

| **Characteristics** | **Number of patients (percent)** |
| --- | --- |
| **Age** |  |
| <=70 | 41 (56.2%) |
| >70 | 32 (43.8%) |
| **Disease-specific survival status** |  |
| Alive | 38 (52.1%) |
| Dead | 35 (47.9%) |
| **Overall survival status** |  |
| Alive | 28 (38.4%) |
| Dead | 45 (61.6%) |
| **Total** | 73 (100%) |

**Table S5. Clinical characteristics in the TCGA-BLCA cohort**

| **Characteristics** | **Number of patients (percent)** |
| --- | --- |
| **Gender** |  |
| Male | 290 (73.6%) |
| Female | 104 (26.4%) |
| **Age** |  |
| <=70 | 226 (57.4%) |
| >70 | 168 (42.6%) |
| **Tumor stage** |  |
| Stage I | 2 (0.5%) |
| Stage II | 124 (31.5%) |
| Stage III | 138 (35.0%) |
| Stage IV | 130 (33.0%) |
| **T stage** |  |
| T0 | 1 (0.3%) |
| T1 | 3 (0.8%) |
| T2 | 112 (28.4%) |
| T3 | 190 (48.2%) |
| T4 | 57 (14.5%) |
| NA | 31 (7.8%) |
| **N stage** |  |
| Nx | 36 (9.0%) |
| N0 | 228 (57.9%) |
| N1 | 44 (11.2%) |
| N2 | 74 (18.8%) |
| N3 | 7 (1.8%) |
| NA | 5 (1.3%) |
| **M stage** |  |
| MX | 193 (49.0%) |
| M0 | 188 (47.7%) |
| M1 | 10 (2.5%) |
| NA | 3 (0.8%) |
| **Overall survival status** |  |
| Alive | 219 (55.6%) |
| Dead | 175 (44.4%) |
| **Total** | 394 (100%) |

**Table S6. The sequences of primers used in this study**

| **Name** | **Primer sequence** |
| --- | --- |
| β-actin | forward: 5'-CACCATTGGCAATGAGCGGTTC-3' |
|  | reverse: 5'-AGGTCTTTGCGGATGTCCACGT-3' |
| ANXA2 | forward: 5'- GAGCGGGATGCTTTGAACATT -3' |
|  | reverse: 5'- TAGGCGAAGGCAATATCCTGT -3' |
| GALK1 | forward: 5'- TCGGTGGGCCAACTATGTC -3' |
|  | reverse: 5'- GCAGCTATTGTGCCCGAGT -3' |
| COL5A1 | forward: 5’-TACCCTGCGTCTGCATTTCC-3’ |
|  | reverse: 5’-GCTCGTTGTAGATGGAGACCA -3’ |
| HS3ST1 | forward: 5’-TTTCACGTCGCCCAAAGTG-3’ |
|  | reverse: 5’-TGGGTGTAGTCAGATAGCACG-3’ |

**Table S7. Characteristics of hypoxia-related genes screened by the random survival forest algorithm**

| **Variable** | **Importance** | **Depth** | **Discription** |
| --- | --- | --- | --- |
| FAM162A | 0.0030 | 2.788 | family with sequence similarity 162 member A |
| ILVBL | 0.0025 | 2.917 | ilvB acetolactate synthase like |
| ANXA2 | 0.0060 | 2.921 | annexin A2 |
| CAVIN3 | 0.0019 | 3.574 | caveolae associated protein 3 |
| HS3ST1 | 0.0006 | 3.429 | heparan sulfate-glucosamine 3-sulfotransferase 1 |
| CAVIN1 | 0.0007 | 3.540 | caveolae associated protein 1 |
| COL5A1 | 0.0061 | 2.886 | collagen type V alpha 1 chain |
| GALK1 | 0.0045 | 2.868 | galactokinase 1 |

**Table S8. Receiver operating characteristic analysis for evaluating the accuracy of the DSS prediction by risk score and clinical characteristics**

| **Datasets** | **Characteristics** | **3-year AUC** | **5-year AUC** |
| --- | --- | --- | --- |
| GSE32894 | risk score | 0.869 | 0.848 |
|  | tumor grade | 0.779 | 0.780 |
|  | tumor stage | 0.762 | 0.764 |
| GSE32548 | risk score | 0.760 | 0.776 |
|  | age | 0.446 | 0.440 |
|  | tumor grade | 0.828 | 0.770 |
|  | tumor stage | 0.625 | 0.651 |
| GSE13507 | risk score | 0.838 | 0.858 |
|  | age | 0.683 | 0.697 |
|  | tumor stage | 0.571 | 0.605 |
| GSE48075 | risk score | 0.662 | 0.631 |
|  | age | 0.594 | 0.595 |
|  | tumor stage | 0.637 | 0.664 |

**Table S9. Immunhistochemical staining levels in 14 bladder cancer tissues by using german immunoreactive score**

| **Protein** | **Riskgroup** | **Negative/weakly positive** | **Moderately positive** | **Strongly positive** |
| --- | --- | --- | --- | --- |
| ERBB3 | Low risk | 3 | 3 | 1 |
|  | High risk | 3 | 2 | 2 |
| FGFR3 | Low risk | 6 | 1 | 0 |
|  | High risk | 7 | 0 | 0 |
| CTLA4 | Low risk | 3 | 4 | 0 |
|  | High risk | 3 | 0 | 4 |
| CD274 | Low risk | 7 | 0 | 0 |
|  | High risk | 7 | 0 | 0 |
| PDCD1 | Low risk | 7 | 0 | 0 |
|  | High risk | 7 | 0 | 0 |
| LAG3 | Low risk | 6 | 1 | 0 |
|  | High risk | 4 | 4 | 0 |
